# Single institution retrospective study evaluating the frequency of implant removal and associated risk factors following open fracture fixation in 80 cases (2010–2020)

**DOI:** 10.1186/s12917-023-03687-0

**Published:** 2023-08-10

**Authors:** Brea M. Sandness, Karen L. Perry, Mieghan Bruce

**Affiliations:** 1 Department of Small Animal Clinical Sciences, Michigan State University, 736 Wilson Road, East Lansing, MI 48824–1314 USA; 2 School of Veterinary Medicine and Centre for Biosecurity and One Health, Murdoch University, Murdoch, 6150 Australia

**Keywords:** Open fracture, Canine, Feline, Complications, Explantation, Infection

## Abstract

**Background:**

Open fractures occur commonly in small animals and are characterised by contamination of the fracture site. While never quantified, it is believed that open fractures stabilised with internal implants are at a higher risk for requiring explantation. This retrospective study determines the frequency and risk factors for explantation following use of internal fixation. Medical records of client-owned dogs and cats with an open fracture, between 2010 and 2020 stabilised using internal implants, were included. Data retrieved included signalment, cause and characterisation of the fracture, comorbidities, preexisting infections, and all details related to anesthesia and surgery. Pre-, Peri- and post-operative antibiotic use were detailed. All cases were followed to clinical union. Postoperative complications, including requirement for implant removal were recorded and classified as major or minor. Associations between potential risk factors and need for explantation were assessed.

**Results:**

Of 80 cases, 72 (90%) were dogs and eight (10%) cats. Major complications were encountered in 23 (28.75%) cases and minor complications in 16 (20%) cases. Explantation was performed in 17 cases (21.25%). Out of 72 dogs, 13 required explantation (18%) whereas four of the eight cats needed implants removed (50%). Only diagnosis of postoperative infection was associated with an increased risk of explantation (RR 2.77; 95% CI 1.25; 6.15; p = 0.045).

**Conclusion:**

Approximately 1 in 5 open fractures stabilised using internal fixation can be anticipated to require explantation, with cats potentially being at a higher risk than dogs. Cases diagnosed with postoperative infection are at a higher risk for requiring implant removal.

**Supplementary Information:**

The online version contains supplementary material available at 10.1186/s12917-023-03687-0.

## Introduction

Open fractures occur commonly in dogs and cats representing 14% and 29% of traumatic fractures in these species respectively [1]. An open fracture is defined as any fracture accompanied by a break in the skin that communicates with the fracture or its associated hematoma. They are characterised by contamination of the fracture site with microorganisms and sometimes introduction of foreign bodies into the wound [2]. Healing potential can be affected by the host’s defence mechanisms to these contaminating microorganisms as well as via damage to the surrounding soft tissue envelope which may impact on bone vascularity. As a result, open fractures are associated with an increased risk of complications including infection and nonunion, presenting a therapeutic challenge [3]. It is a general belief that following fracture stabilisation with internal implants, open fracture patients are at a higher risk for requiring explantation than patients with closed fractures. However, this proposed increased risk has never been quantified in small animal patients.

Controversy regarding the most appropriate method of fracture stabilisation for open fractures abounds in both the human and veterinary literature. It is a common misconception that open fractures preclude the use of internal fixation. Prospective randomised studies comparing internal and external fixation for treatment of open diaphyseal fractures in people demonstrated no differences in fracture-site infection or bone healing rates between the two methods [4, 5]. In fact, recent work indicates that definitive treatment with modern external ring fixators results in a higher probability of at least one major limb complication when compared to internal fixation [6]. Stability at the fracture site prevents further injury to the soft tissues and enhances the host response to contaminating organisms [7]. As such, a general principle in selecting the approach and fixation method is to choose the technique that provides fracture stability while allowing access to the wound and preserving the integrity of the remaining viable soft tissues [8, 9]. Open fractures in veterinary patients have been successfully repaired with both internal fixation methods (bone plates, plate-rod technique, cerclage wires and interlocking nails), and external skeletal fixation including linear, circular and hybrid fixators [10–18] but detailed follow-up remains sparse.

When discussing open fracture management, pet owners are frequently concerned regarding several aspects of care including their pet’s welfare, total duration of management, cost, number of veterinary visits involved, requirements for future surgeries, and likelihood of returning to an acceptable level of function. Unfortunately, despite the common nature of open fractures, there is a paucity of evidence in the peer-reviewed small animal literature prompting extrapolation from human literature and reliance upon veterinarian experience to answer such queries. More evidence specific to the small animal field is certainly required. As such, the objectives of this study were to determine the frequency of, and risk factors for, explantation following the use of internal fixation to stabilise open fractures.

## Results

Of 93 records initially identified and evaluated, 64 had documented follow-up to clinical union within existing records. For 16 cases, telephone follow-up with the owner or primary care veterinarian was undertaken to determine whether complications had occurred, and 13 were excluded due to a lack of follow-up extending to clinical union of the fracture.

### Demographic variables

Of the 80 cases included, 72 (90%) were dogs and eight (10%) were cats. One dog had two open fractures repaired 23.5 h apart. Thirty-four dog breeds were represented, the most common.

being mixed breed (n = 16), Labrador retriever (n = 9), German shepherd (n = 6) and goldendoodle (n = 3). There were two each of boxer, German shorthair pointer, golden retriever, American pit bull terrier, Shetland sheepdog, Siberian husky, and Yorkshire terrier with one each of the remaining breeds. Within the feline cohort, seven (87.5%) were domestic short hair and one (12.7%) was a domestic long hair.

Body condition score (BCS) was reported for 72 cases with a median of 5/9. Most animals (74%) were classified as ideal weight, 14 with BCS 4/9, 39 with 5/9. Thirteen animals (18.1%) were overweight, ten with BCS 6/9, three with BCS 7/9, and six (8.3%) were obese, four with BCS 8/9 and two with BCS 9/9. The median patient age was 3.0 years (range 7 weeks − 11 years). There were 41 (51.2%) females of which 33 (80.5%) were spayed, and 39 (48.8%) males of which 27 (69.2%) were neutered.

In 42 cases (52.6%) the source of open fracture was a road traffic accident. Eight cases (10%) were attacked by another animal or person, seven cases (8.9%) suffered the injury because of jumping or falling and in 12 cases (15.0%) the cause of injury was unknown. Other causes included gunshot-induced fractures (n = 4, 5%), running into stationary objects (n = 4, 5%), getting stuck on a stationary object (n = 2, 2.6%), and sustaining the fracture whilst running (n = 1, 1.3%).

Thirty-seven cases (46.2%) had at least one co-morbidity; eleven cases had two co-morbidities and one case had three. Thoracic trauma was the most commonly encountered co-morbidity representing 30% of co-morbidities. Additional co-morbidities are detailed in Table 1. Seven cases (8.8%) had a history of pre-existing infection. Three cases had an active ectoparasite infection, one had a urinary tract infection, one had active pyoderma, and two others had Lyme disease.

**Table 1:** Traumatic co-morbidities encountered in 37 cases with open fractures. Remaining 30 cases had no co-morbidities

Co-morbidity	Number of cases
Pulmonary contusions	6
Anemia	5
Hemoabdomen	4
Soft tissue wounds remote from fracture site	4
Pneumothorax	3
Thrombocytopenia	3
Regurgitation	3
Hemothorax	2
Arrhythmia	2
Brachial plexus injury	2
Pneumomediastinum	1
Rib fractures	1
Traumatic intervertebral disc herniation	1
Vertebral fracture	1
Horner’s Syndrome	1
Sacro-coccygeal luxation	1
Traumatic brain injury	1
Urticaria	1
Brachycephalic Obstructive Airway Syndrome	1
Aspiration pneumonia	1
Hematochezia	1
Heart murmur	1
Diabetes Mellitus	1
Soft tissue sarcoma (remote from fracture)	1
Azotemia	1
Hepatopathy	1

### Fracture variables

Regarding open fracture characteristics, 44 (55%) animals suffered right-sided open fractures, 34 (42.5%) left-sided and two (2.5%) suffered bilateral open fractures. Forty-eight animals (60%) suffered open fractures affecting a hind limb, 26 (32.5%) suffered an open fracture of the forelimb, four (5.0%) suffered injuries to both a forelimb and a hind limb, one (1.3%) suffered an open pelvic fracture and one (1.3%) sustained both pelvic and hind limb open fractures. Fractures were comminuted in 61 (76.2%) animals, and simple in 19 (23.8%). Details regarding bone location and distribution are detailed in Table 2. The majority of cases sustained fractures proximal to the carpus/tarsus and distal to elbow/stifle (n = 47, 58.8%) with fractures of the tibia/fibula making up the majority of these cases (33.8%).

**Table 2:** Table demonstrating the distribution of open fractures by location

Limb Site	Bone	Number of cases (N)	Incidence
**Proximal** (Proximal to elbow/stifle) **N = 21** **26.2%**	Femur	13	16.2%
	Humerus	8	10%
**Middle** (Proximal to carpus/tarsus but distal to stifle/elbow) **N = 47** **58.8%**	Tibia & Fibula	27	33.8%
	Radius & Ulna	20	25.0%
**Distal** (At the level of the tarsus/carpus and distally) **N = 8** **10%**	Metabone	5	6.2%
	Other	3	3.8%
**Multiple Limbs**		**4**	**5%**

### Antibiotic use

Information collected regarding antibiotic use is detailed in Tables 3 and 4. Perioperative antibiotics were used in all cases with cefazolin (n = 35, 43.8%) and ampicillin-sulbactam (n = 35, 43.8%) being the most common. The first dose of antibiotic therapy was administered at a median of 255 min (range 26 to 30,200 min) following injury. Post lavage aerobic or anaerobic cultures were collected in eighteen patients (22.5%). Seventy-nine patients received postoperative antibiotics with potentiated-amoxicillin (n = 44, 55%) and cephalexin (n = 31, 38.8%) being the most commonly prescribed. Duration of postoperative antibiotic use (Table 4) ranged from ≤ 7 days to > 42 days with the longest course being 21 weeks. The median duration of antibiotics for all patients was two weeks (range of 0 to 17 days). The median duration of antibiotics for those that required explantation was 2.14 weeks (range of 0.71 to 17 weeks).

**Table 3:** Type and frequency of antibiotics administered perioperatively and postoperatively along with incidence for explantation

Type of antibiotic		Number of cases	Incidence	Incidence of explantation
**Perioperative**	Cefazolin	35	43.75%	10 (28.6%)
	Ampicillin-sulbactam	35	43.75%	6 (17.1%)
	Ampicillin	6	7.5%	1 (16.7%)
	Cephalexin	2	2.5%	0 (0%)
	Clavamox	1	1.25%	0 (0%)
	Ampicillin-sulbactam & Enrofloxacin	1	1.25%	0 (0%)
**Postoperative**	Clavamox	44	55%	8 (18.2%)
	Cephalexin	31	38.75%	7 (22.6%)
	Clavamox & Enrofloxacin	2	2.5%	1 (50%)
	Amoxicillin	1	1.25%	0 (0%)
	Doxycycline	1	1.25%	1 (100%)
	None	1	1.25%	0 (0%)

**Table 4:** Duration of antibiotic therapy prescribed post-operatively in 80 cases following internal fixation of open fractures

Length of Course of Antibiotics	Number of cases	Incidence
≤ 7 days	8	10%
≤ 14 days	44	55%
≤ 21 days	14	17.5%
≤ 28 days	2	2.5%
≤ 35 days	0	0%
≤ 42 days	2	2.5%
≥ 42 days	5	6.3%
Unknown length	5	6.3%

### Operative variables

Median time from trauma until surgery was 74.4 h (range 4.0 to 672 h). Median duration of surgery for fixation and associated general anaesthesia was 135 min (range of 50–430 min) and 260 min (range of 110–635 min) respectively. If additional anaesthesia was needed for wound care prior to or following surgery this was included in total anaesthestic time within the hospitalisation period. Most cases underwent only one anaesthetic event (n = 68, 85%). However, 10 cases (12.5%) underwent two anaesthetic events and two cases (2.5%) underwent three anaesthetic events either for revision surgery, fracture fixation on another limb or wound care. Median for total duration of anesthesia including these events was 263 min (range of 110–850 min).

Sixty-five fractures (81.25%) were repaired using an open approach while minimally invasive techniques were performed in 15 fractures (18.75%). The most common stabilisation method used was isolated plate fixation in 42 (52.5%) cases. Angle-stable interlocking nails were used in 19 (23.75%) cases, plate-rod technique in 12 (15.0%) cases, Kirschner wires alone in three (3.75%) cases and other internal fixation methods in four (5.0%) cases. Primary closure of the open fracture wound was performed in 68 (85.0%) cases, open wound management and healing by second intention was elected in eight (10.0%) cases, partial closure leaving the remainder to heal by second intention was chosen in one (1.25%) case and one case (1.25%) underwent negative pressure wound therapy prior to delayed primary closure. Additionally, two cases (2.5%) were treated chronically, such that their open fracture wounds had healed via second intention prior to surgical intervention. This rendered 68 cases being classified as primary closure and 12 cases being classified as other. Bupivacaine liposome injectable suspension (Nocita®) was used in three (3.75%) cases. Skin staples were used in 46 (57.5%) cases.

### Postoperative variables

Complications were encountered in 34 (42.5%) cases which are detailed in Table 5. Major complications were encountered in 23 (28.75%) cases and minor complications in 16 (20%) cases. There were both minor and major complications in five cases (6.25%). Outpatient wound/incision-related complications were the most common minor complications whereas, deep surgical site infections (SSI) were the most common major complication.

**Table 5:** Details of minor and major complications encountered in 16 cases (20%) and 23 cases (28.75%) respectively presenting with open fractures. Five cases (n = 5, 29.4%) sustained both a minor and major complication that required implant explantation

Complications		Number of cases
**MINOR**	Outpatient open wound management	8
	Implant failure not necessitating revision surgery	3
	Incisional dehiscence	2
	External coaptation associated morbidity	2
	Neuropraxia	1
**MAJOR**	Deep SSI; Pseudomonas aeruginosa (1), Escherichia coli & Enterobacter cloacae (1), E. coli, Corynebacterium species, Enterococcus faecalis, Aeromonas species, growth of Proteus species (1) unknown (4)	7
	Implant failure (5)/ Implant exposure (1)	6
	Improper implant placement necessitating immediate revision	3
	Osetomyelitis; Pseudomonas aeruginosa (1), Escherichia coli & Enterococcus species (1)	2
	Bone sequestrum	1
	Avascular necrosis of the surgical limb	1
	Contralateral hip reluxation in a polytrauma case following initial open reduction	1
	Osteosarcoma affecting same limb segment 9 years postoperatively	1
	Skin necrosis	1

Median follow-up time was 12 weeks (range 2.1 to 572 weeks). This included 12 skeletally immature (≤ 10 months of age) cases that achieved clinical union between 2 and 8 weeks postoperatively which explain the lower values within this data range. One case, a three-year-old domestic shorthaired cat, suffered avascular necrosis of the limb and underwent an amputation following initial fracture stabilisation. This case was included as a major complication and within the explant group but was the only case included that did not have follow-up to clinical union.

Implant removal was performed in 17 of the 80 cases (21.25%). Out of 72 dogs, 13 required explantation (18%) whereas four of the eight cats needed their implants removed (50%). Indications for implant removal are detailed further in Additional file 1; in 11 cases (64.7% of those explanted), implants were removed due to the presence of recurrent draining tracts, osteomyelitis, implant-associated infection or implant exposure. In five cases (29.4% of those where implants were removed), explantation was performed due to implant failure, breakage or migration and in one case (5.9% of those explanted) due to avascular necrosis of the limb. Of the 17 cases that were explanted, two (11.8%) of them had a history of pre-existing infection; one case had fleas and the other a history of Lyme disease. One canine patient was diagnosed with, and treated aggressively for, a severe deep SSI. Amputation was recommended however the owners elected for humane euthanasia 17 days postoperatively. This case was included in the major complications group. Additional information regarding each of these cases including details of culture results and revision surgeries where performed are available in Additional file 1.

### Statistical analysis

Univariable analysis showed that seven variables (Additional file 2) had a P < 0.25 and met the criteria for inclusion in the multivariable analysis. Based on this analysis, the risk of explantation was higher in cats than in dogs, (RR 2.77, 95% CI 1.18–6.48, P = 0.075) and higher in neutered cases than in intact cases (RR 5.33, 95% CI 0.75–37.71, P = 0.104). Being overweight was associated with a higher risk for explantation than being of ideal BCS (RR 1.95, 95% CI 0.87–4.40, P = 0.174) as was the presence of co-morbidities (RR 2.13, 95% CI 0.87–5.20, P = 0.092). Fracture site was associated with explantation with proximal limb sites being less likely to require implant removal than middle or distal sites (RR 0.34, 95% CI 0.09–1.39, P = 0.16). Cases where a longer course of postoperative antibiosis was prescribed had an increased risk of explantation (RR 1.13, 95% CI 1.08–1.19, P = 0.046), and finally, cases diagnosed with a post-operative infection were at higher risk for explantation than those where no such diagnosis was made (RR 2.77, 95% CI 1.25–6.15, P = 0.045).

### Multivariable assessment

These seven variables were included in the multivariable logistic regression model. The final model revealed that only the diagnosis of postoperative infection was associated with an increased risk of explantation. There was no evidence of other variables being associated with the risk of implant removal.

## Discussion

This study aimed to determine the frequency of explantation following internal fixation of open fractures in dogs and cats and explore potential risk factors for explantation at a single referral center over a 10-year period. Explantation was performed in 21.25% of open fractures stabilised using internal fixation techniques. This is substantially higher than the reported implant removal rates of a closed procedure, such as, an elective tibial plateau leveling osteotomy where 3.0 -7.4% of cases required explantation [19–21]. This explantation rate is also higher than that reported in previously published case series detailing fracture stabilisation where 14.6–17.0% of cases required implant removal [22–24]; however, it should be considered that such case series do include open fractures comprising up to 12% of the study population [24]. This relatively high frequency of implant removal warrants consideration during preoperative planning for open fracture stabilisation; knowing that approximately 1 in 5 open fractures stabilised with internal fixation will require implant removal, the surgeon should ensure that implants are placed in a manner that will facilitate explantation should this be required. 18% of dogs required explantation whereas 50% of cats had their implants removed. While this difference was only statistically significant during univariable analysis and the total number of cats in the study was low, it may indicate cats to be at a greater risk of explantation than dogs. When looking at the feline fractures that required explantation in Additional file 1, all were tibia/fibular fractures so these may be at particular risk. This correlates with literature in humans where the infection rate for open tibial fractures is twice that for open fractures in other locations [25, 26]. While further studies are indicated to investigate this further, potential explanations for this may include the minimal soft tissue envelope in the feline extremities.

The overall complication rate in this retrospective study was 42.5% with major complications being encountered in 28.75% of cases. There are no previous reports detailing complication rates following open fracture management in veterinary patients. Open fractures are expected to be associated with increased risks of complications such as infection, delayed union and nonunion due to contamination and damaged blood supply [8, 27–31]. As such, it is not surprising that the complication rate reported here exceeds that previously reported for either closed fractures or elective orthopedic procedures. Additional file 1 describes the reasons for implant removal in the 17 cases where this was necessary. Five cases (29.4% of those explanted) required explantation due to implant failure/breakage/migration, while 11 (64.7% of those explanted) were due to recurrent draining tracts, osteomyelitis, implant-associated infection or implant exposure. The remaining case was a cat that developed avascular necrosis of the limb two weeks following surgery. Therefore, although 21.25% of cases required implant removal, only 13.8% of cases were explanted due to the presence of infection. While this remains more than double the infection risk following elective, closed orthopedic procedures, this may indicate that efforts to reduce complication rates following open fracture stabilisation need to focus on other areas such as optimising stability, in addition to identifying and avoiding risk factors for SSI.

While antibiotic coverage and appropriate management of soft-tissue injuries have likely helped to reduce the incidence of infection and consequent complications as veterinary medicine has advanced, the risk remains higher for open fractures than for closed injuries. The explantation and complication rates reported herein will help facilitate owner communication, allowing realistic expectations to be articulated regarding the potential complexity of recovery following open fracture management, and specifically, the prospective requirement for a second surgical procedure.

The only variable that remained associated with an increased risk of explantation after multivariable analysis was a diagnosis of postoperative infection showing that animals who suffer postoperative infection have an increased risk of explantation. People who suffer injuries complicated by infection have also been shown to be more likely to require further operative management [32]. This indicates that, while not the only factor to consider, reducing post-operative infection should remain a focus of prevention strategies when managing open fractures. In addition to appropriate systemic antibiotic administration, other strategies which may deserve further attention to decrease infection include use of local antibiotic therapies and the antimicrobial functionalising of implant surfaces to avoid formation of biofilm [33, 34].

Early antibiotic administration remains a key principle of open fracture management because most patients with open fractures have contaminated wounds [25, 27]. This early antibiotic administration is considered to be important as delayed administration of the first dose of antibiotic prophylaxis in humans has been shown to increase the risk of infection markedly [26]. A retrospective human study evaluating 137 type III open tibial fractures showed that administration of antibiotics beyond 66 min from injury was an independent risk factor for infection with an odds ratio of almost four [35]. In the current study, median time from injury to first antibiotic administration was 4 h 15 min and, interestingly, increasing durations of time between injury and first antibiotic administration showed no increased risk for explantation.

While early and perioperative antibiotic administration are considered the standard of care, there is still controversary regarding the optimal length of postoperative antibiotic therapy. Evidence in the human field suggests that antibiotics should continue to be administered until primary closure of the wound, or for a total of 72 h, whichever is sooner [36, 37]. This contrasts markedly with common practice in the veterinary field where protracted courses of antibiotics are often prescribed. In this study, the median duration of postoperative antibiotics prescribed was 14 days (Table 4). Statistical analysis did not reveal any evidence that longer courses of antibiotics were associated with lower rates of explantation, in fact, if anything, based on the univariable analysis, patients undergoing explantation had longer courses of antibiotics than those which did not. The interpretation of this is challenging as there are likely a plethora of reasons behind the decision to prescribe a longer or shorter antibiotic course which are not discernible from medical record review; it may be that the cases where longer antibiotic courses were prescribed were those that also had wounds that required ongoing treatment for example. Additionally, without having information on the open fracture classification, it is possible that the duration of antibiotics is a proxy for underlying associations, rather than being directly associated with explantation itself. More detailed studies investigating the rationale for both choice of antibiotic and duration of antibiotic course are considered warranted.

As detailed within the methodology, due to the retrospective nature of this study and the very inconsistent reporting of open fracture classification within the medial records, the decision was made not to attempt classification via the Gustilo-Anderson system [27, 38]. Consideration was given to attempting to extrapolate from the information available within the medical record, however, given that only moderate (60%) interobserver agreement is reported with classification based on case evaluation and videotaped case presentations [39, 40], reliability of grading based on written medical record review was considered to be highly questionable. Additionally, given the prognostic relevance of soft-tissue and bone injury in the depths of the open fracture wound, it is recommended that fractures be classified not in the emergency room, but in the operating room after surgical exploration and debridement have been completed [41]. As one of the aims of this study was to facilitate owner communication and deliver accurate expectations when encountering these severe injuries, the authors consider that knowledge of the overall explantation rate for open fractures, regardless of classification, represents useful information, especially as most of these conversations will take place prior to having an accurate fracture classification available.

This study did not reveal evidence that the type of antibiotic used had an effect on the risk for explantation. Although in the past cultures were routinely performed both before and after debridement of the open fracture wound, authors of many studies in the human field have questioned their utility [42–45]. Lee studied pre debridement cultures and found that only 8% of 226 organisms grown on culture eventually caused infection and 7% of 106 patients with negative cultures eventually became infected [42]. Post-debridement cultures were not much better as only 25% of 32 organisms grown on culture eventually caused infection and 12% of 86 patients with negative cultures eventually became infected [42]. Hamil et al. [45] additionally investigated the use of quantitative cultures but even these were not predictive for either infection occurring, nor which organism would cause infection. The organisms that are found to be contaminating an open fracture on presentation do not represent the microbes that will eventually cause infection. In fact, there is evidence that most infections at the site of open fractures are caused by nosocomial bacteria [44]. As such, wound cultures are not routinely taken at the authors’ facility unless an active infection is present. As most open fracture wounds are caused by gram-negative rods and gram-positive staphylococci [27, 38, 42, 44], systemic antibiotic choice is directed toward a wide-range of Gram-positive and Gram-negative organisms with cefazolin and ampicillin-sulbactam being the two most commonly used on initial presentation.

While these results are interesting, due to the limitations of this retrospective study the findings pertaining to antibiotic choice and duration of therapy should be interpreted with caution, with decisions being based on individual clinical patient assessment. However, shorter courses of antibiotics may be indicated in some open fracture patients, more in line with treatment recommendations within the human medical field.

In this study, the time between sustaining the open fracture and proceeding to surgery varied greatly as in some cases the pet had been missing for several days and returned with an injury. Statistical analysis did not reveal any correlation between the time taken to proceed to surgery and the risk for explantation. This provides evidence that open fractures do not need to be considered as surgical emergencies. Considering 46.2% of cases in this study had at least one comorbidity, our results indicate that it is entirely acceptable to focus on appropriate management and stabilisation of such co-morbidities with patients only proceeding to surgery when systemically stable and when the apposite team is present to facilitate surgical management. Appropriate emergency management of the open fracture is still warranted in the interim however including administration of systemic antibiotics, thorough irrigation, debridement as necessary and coverage of the wound.

### Limitations

There are several important limitations to our study that deserve consideration. Our primary aim was to determine the frequency of explantation and any risk factors associated with that and as such, the statistical model was designed accordingly. This limited the investigation of associations between other variables as they were considered principally as confounding factors.

The retrospective nature of the study introduces many potential sources of error, particularly with regard to the potential for reporting inaccuracies and the lack of standardisation. Some of the effects of this will have been reduced by the reporting of a large number of cases and only including cases from a single institution but they should still be considered. Particularly, while the study includes a large number of cases there is still a low number of cats in the study, which limits the conclusions that can be made.

A significant limitation is that fracture classification was not included within our analysis and as such could not be analysed as a risk factor. Although this was initially planned, upon review of medical records it became clear that many open fractures had not been classified, neither upon initial presentation nor intraoperatively. The most commonly used classification scheme for open fractures currently in the veterinary field remains the Gustilo-Anderson classification [27, 38]. In humans, this classification has been shown to determine the risk of infection which ranges from 0 to 2% for type I open fractures, 2–10% for type II and 10–50% for type III [26, 38]. As mentioned previously, the major limitation of this system is that the inter-observer reliability has been shown to be poor with only moderate (kappa 0.59) and average agreement of 60% [39, 40]. While the initial plan for this study did include fracture classification, the validity of such classification based on medical record review was considered likely to result in such poor reliability that it would potentially be more misleading than clinically useful. As such, this was not pursued. Based on our experience during data collection for this study, and despite the recognised limitations of current classification schemes, the consistent recording of open fracture classification should be encouraged at time of presentation and at the time of surgery in order to facilitate future research efforts. It is the intention of the authors to use the data included in this report to serve as a foundation for future prospective studies in which grading will be performed utilising various grading systems. This may assist determination of whether any classification system can be of use in predicting complications or patient outcomes.

## Conclusion

In conclusion, this study demonstrated that 21% of open fractures stabilised using internal fixation can be anticipated to require explantation with 65% of these being due to infection. Cats may potentially be at a higher risk than dogs. Thus, approximately one in five open fractures stabilised with internal implants will require a second procedure for implant removal and preoperative planning should take this into account. A diagnosis of postoperative infection renders explantation more likely to be required, and as such, efforts should be focused on the prevention of infection throughout case management. This novel information can be used to provide more accurate prognoses to clients and to manage client expectations.

## Materials and methods

The database at a single specialty academic referral center was searched for client-owned dogs and cats diagnosed with an open fracture that was stabilised using internal implants between October 2010 and August 2020. Fractures stabilised using external skeletal fixation or external coaptation, or cases lacking follow-up to clinical union were excluded.

Data obtained from the medical record included patient species, breed, sex, age, weight, body condition score, on a 9-point scale as defined by Laflamme et al. [46], and cause of fracture. Fractures were classified by bone(s) affected (defined as metabones, radius/ulna, tibia/fibula, humerus, femur, other or multiple). Additional classification was based upon location along the limb as distal (from tarsus/carpus distally), middle (proximal to carpus/tarsus but distal to stifle/elbow), proximal (proximal to elbow/stifle) or multiple. Fractures were also classified as simple or comminuted. The grade of each fracture was excluded from consideration as it was not consistently present within the medical records. Given that only moderate (60%) interobserver agreement is reported with classification based on case evaluation and videotaped case presentations [39, 40], an attempt to retrospectively classify fractures using the Gustilo-Anderson scheme [27, 38], based on information within the medical record, was considered likely to be more misleading than productive.

Cases were classified as polytraumatic if additional orthopedic injuries were diagnosed at the time of open fracture management. Additional non-orthopedic co-morbidities were recorded and specifically, any known history of infection was detailed.

All available details related to antibiotic use were recorded. Specifically, the time between patient presentation and commencement of broad-spectrum antibiotics; the type of perioperative antibiotics used; and the type and duration of postoperative antibiotics used were documented.

The time between sustaining the open fracture and surgical stabilisation was recorded. Specific to the surgical procedure, anaesthesia time, surgical time and number of anaesthetic episodes were detailed. The approach was defined as minimally invasive osteosynthesis (MIO) or open reduction and internal fixation (ORIF) and the type of fixation was defined as either plate fixation in isolation, plate-rod construct, angle-stable interlocking nail, Kirschner wires or other. The use of bupivacaine liposome injectable suspension (Nocita®) (yes/no), skin staples (yes/no), collection of post lavage aerobic or anaerobic cultures (yes/no), need for additional wound care following fracture stabilisation (yes/no) and placement of external coaptation postoperatively (yes/no) were noted. Specifically, how the open fracture wound was managed was also recorded and classified as primary closure, open wound management with healing by second intention, partial closure with the remainder healing by second intention or use of negative pressure wound therapy followed by delayed primary closure. For the purposes of statistical analysis, wound management was considered as primary closure or other.

Presence of postoperative complications, including requirement for explantation or amputation was recorded, and the specific details of the complication noted. Complications were defined as any undesirable outcome associated with the surgical procedure and were classified as major (surgical intervention performed) or minor (managed without surgical intervention). The presence of postoperative infection was specifically recorded. A wound was considered infected when purulent discharge, abscess, sinus and/or one or more of the clinical signs of pain and localised swelling, redness, heat, fever, or deep incision spontaneous dehiscence was identified on physical examination and/or when an organism was isolated from an aseptically collected sample by culture and/or a positive cytology study [47, 48]. If revision surgery or explantation was required, then the type of surgery was documented along with the results of any culture samples obtained at the time of the revision surgery.

### Statistical analysis

Continuous data were tested for normality by visual inspection of the histogram and normal quantile plot. Normally distributed continuous data are presented as mean ± SD; non-normally distributed continuous data are presented as median and range. Categorical variables are reported as proportions (%). Univariable Poisson regression was used to evaluate crude association between exposure variables of interest and explantation. The association between weight and explantation was assessed separately for dogs and cats as cats typically weigh less than most dog breeds and thus this is not a biologically meaningful variable when dogs and cats are combined in the same model.

Explanatory variables with a P < 0.25 and any a priori potential confounding factors identified using causal diagrams were included in the multivariable Poisson regression model with robust standard errors to assess for association with explantation. Selected variables were assessed for collinearity, and if found, the most biologically meaningful variable was retained. Variables associated with the highest p-values were eliminated sequentially by backward selection to identify the most parsimonious model, including only variables with P < 0.05 or any confounding variables that caused a change > 20% in the risk ratio (RR). Two-way interactions were examined between explanatory variables and retained when P < 0.05. The goodness of fit for the Poisson regression model was assessed with the deviance test. Statistical analyses were performed using statistical softwarea (R 4.1.3. using packages: sandwich [49], and tidyverse [50].

### Electronic supplementary material

Below is the link to the electronic supplementary material.

Supplementary Material 1: Details of open fractures requiring explantation or amputation (Polytrauma cases have the open fracture highlighted in bold).

Supplementary Material 2: Baseline distribution of risk factors for explantation in open fractures stabilised using internal fixation.

## Data Availability

The datasets used and/or analysed during the current study are available from the corresponding author on reasonable request.
