# Supplementary material for: Single institution retrospective study evaluating the frequency of implant removal and associated risk factors following open fracture fixation in 80 cases (2010–2020)
Source: BMC Vet Res. 2023 Aug 10;19:119. doi: 10.1186/s12917-023-03687-0 (PMC10413701; doi:10.1186/s12917-023-03687-0)
Supplement: Supplementary file 1 — Supplementary Material 1: Details of open fractures requiring explantation or amputation (Polytrauma cases have the open fracture highlighted in bold). [file 12917_2023_3687_MOESM1_ESM.docx]

**Additional File 1 – Table of open fractures requiring explantation or amputation (polytrauma cases have open fracture in bold).**

| **Signalment** | **Fracture** | **Type of fixation** | **Culture and sensitivity performed at original surgery** | **Postoperative wound care or bandaging required?** | **Explantation time and reasoning** | **Culture and sensitivity performed at explantation surgery** |
| --- | --- | --- | --- | --- | --- | --- |
| 4 yo  13.6 kg  FS  Canine  Mixed Breed | **1) Left radius/ulnar fracture**  2) Right tarsal fractures and luxation | 1) ORIF Bone plate/screws  2) Bone plate/screws for arthrodesis | No | Wound over tarsal arthrodesis required a week of wound care for delayed primary wound healing | 5 years postoperative due to plate exposure and draining tract | No |
| 2.5 yo  35.5 kg  FS  Canine  Mixed Breed | Right radius/ulnar fracture | ORIF Bone plate/screws | No | None | 7 months postoperatively due to radiographic osteomyelitis | Yes: Pseudomonas aeruginosa |
| 4 yo  27.5 kg  FS  Canine  Mixed Breed | Right radius/ulnar fracture | MIO Bone plate/screws | No | Incisional dehiscence managed with soft padded bandage | 8 weeks postoperatively due to implant breakage | No |
| 8 yo  40.3 kg  MN  Canine Labrador Retriever | Left tibia/fibular fracture | ORIF Cerclage wire with bone plate/screws | No | Incisional dehiscence causing deep SSI requiring surgical closure after hospitalisation | 25 weeks postoperatively due to infection | Yes: Pseudomonas aeruginosa |
| 9 yo  44 kg  FS  Canine  Mixed Breed | Left tibia/fibula fracture | MIO angle-stable interlocking nail | No | None | 6 weeks postoperatively due to catastrophic implant failure. A larger diameter nail was placed. Explantation of this was not required during study follow-up. | No |
| 2 yo  30.9 kg  MN  Canine Labrador Retriever | 1) Right hip luxation  **2) Left tibia/fibular fracture** | 1) Closed reduction  2) ORIF angle-stable interlocking nail | Yes: Escherichia coli and Enterococcus species | Wound over fracture site managed with wound care and delayed primary wound closure | 4 months postoperatively due to osteomyelitis | Yes: Two different strains of Echerichia coli |
| 2 yo  6.9kg  FS  Feline  DSH | Right tibia/fibular fracture | ORIF IM Pin and bone plate/screws | No | IM pin migration proximally that was reduced via ORIF and placed in soft padded bandage for 2 weeks | 11 weeks postoperatively due to implant migration | No |
| 10 mth  41kg  ME  Canine  Chesapeake Bay Retriever | **1) Left radial fracture with ulnar luxation**  2) Left femoral fracture | 1) MIPO bone plate/screws for radius and closed reduction for ulnar luxation  2) MINO angle-stable interlocking nail in femur | No | Wounds on forelimbs requiring frequent cleaning and changing for 2 weeks and fiberglass splint placed for 2 wks due to ulnar luxation | Required removal of radial implants > 24 months later (exact time unknown based on owner recollection) due to recurrent draining tract | No |
| 2 yo  23kg  MN  Canine  Australian Sheepdog | Left femur | ORIF Cerclage wire with bone plate/screws | No | None | Revision 10 weeks postoperatively due to implant failure and bone sequestrum then a second explantation 12 weeks following revision with bone/plate and screws due to screw loosening | Yes: No growth |
| 6.5 yo  4.6kg  FS  Feline  DSH | **1) Right fibular fracture with tibiotarsal luxation**  2) Left second metatarsal fracture  3) Right metatarsal fractures  4) Right coxofemoral luxation | 1) ORIF with pin and tension band for fibular fracture with MCL repair  2) Splint for left metatarsal fracture  3) Non-surgical treatment of right metatarsal fracture  4) Open reduction of right coxofemoral reduction with capsulorraphy | No | No wound care but right pelvic limb splinted for 6 weeks | 13 weeks postoperatively due to implant exposure of pins from right fibular fracture repair | No |
| 3 yo  5.4kg  FS  Feline  DLH | Right fibular fracture with tarsal luxation | ORIF with pin and tension bands along with prosthetic ligaments | No | Bivalve cast for 6 days then switched to soft padded bandage for one week | Amputation 14 days postoperatively due to avascular necrosis of limb | No |
| 5 yo  28kg  FS  Canine  Boxer | Left tibia/fibular fracture | OBDNT IM pin and bone plate/screws | No | None | Revision 3 weeks postoperatively due to implant failure. Revised with IM pin removal and placement of larger bone plate/screws and cancellous autograft. Explantation of these implants was not required during study follow-up. | No |
| 4 yo  11.8kg  MN  Canine  Basenji | Right humeral fracture | ORIF Orthogonal bone plating and lag screw | No | Wounds on forelimbs left to heal by second intention after 6 days of wound care in hospital | Revision 21 weeks postoperatively to remove loose screws with complete explantation 52 weeks post operatively due to implant exposure | Yes: Staphylococcus pseudintermedius |
| 10 yo  6.7kg  FS  Feline  DSH | Right tibia/fibular fracture | ORIF Tarsal arthrodesis | Yes: Escherichia coli & Enterobacter cloacae complex | Surgical debridement of wounds on surgical limb. | Amputation performed due to continued infection | No |
| 5 yo  31.5kg  FS  Canine  Mixed Breed | Left tibia/fibular fracture | ORIF Bone plate/screws | No | Incisional dehiscence managed with outpatient bandages | 8 months postoperatively due to exposed implant and recurrent draining tracts | Yes: Staphlococcus pseudintermedius (MRSP) |
| 10 yo  12kg  MN  Canine  Mixed Breed | Right tibia/fibular fracture | ORIF bone plate/screws and hemi-cerclage | No | Minor incisional irritation managed with soft padded bandage for 1 week | 12 months postoperatively due to recurrent draining tract | Yes: Enterococcus faecium |
| 1 yo  30.6kg  MN  Canine  German Shepherd | Right tibia/fibular fracture | MIPO bone plate/screw | No | No wounds but soft padded bandage placed for 1 week postoperatively | Revision 1 day post operatively due to intra-articular screw and complete explantation 17 months postoperatively due to osteomyelitis | Yes: Staphylococcus aureus |

FS: Female spayed, M: Male intact, MN: Neutered Male, yo: years old, mth: months old, kg: Kilograms, DSH: Domestic short hair, DLH: Domestic long hair, MIO: Minimally invasive osteosynthesis, MINO: Minimally invasive nail osteosynthesis, MIPO: Minimally invasive plate osteosynthesis ORIF: Open reduction and internal fixation, IM: Intramedullary pin, MCL: Medial collateral ligament, SSI: Surgical site infection
