# Supplementary material for: Single institution retrospective study evaluating the frequency of implant removal and associated risk factors following open fracture fixation in 80 cases (2010–2020)
Source: BMC Vet Res. 2023 Aug 10;19:119. doi: 10.1186/s12917-023-03687-0 (PMC10413701; doi:10.1186/s12917-023-03687-0)
Supplement: Supplementary file 2 — Supplementary Material 2: Baseline distribution of risk factors for explantation in open fractures stabilised using internal fixation. [file 12917_2023_3687_MOESM2_ESM.docx]

**Table 6 – Baseline Distribution of Risk Factors for Explantation in Open Fractures stabilized with Internal Fixation.**

|  |  | **Total number of cases**  **(n=80)** | **Number (%) that underwent implant explantation**  **(n=17)** | **RR** | **95% CI** | **P-Value** |
| --- | --- | --- | --- | --- | --- | --- |
| **Species** | Canine | 72 | 13 (18.1%) |  |  |  |
|  | Feline | 8 | 4 (50%) | 2.77 | (1.18, 6.48) | 0.075 |
| **Age (years)** |  |  | 4.0 (0.83; 10)^a^ | 1.05 | (0.45, 2.46) | 0.483 |
| **Sex** | Female | 41 | 11 (26.8%) |  |  |  |
|  | Male | 39 | 6 (15.4%) | 0.57 | (0.25, 1.34) | 0.273 |
| **Neuter status** | Intact | 20 | 1 (5%) |  |  |  |
|  | Neutered | 60 | 16 (26.7%) | 5.33 | (0.75, 37.71) | 0.104 |
| **Weight (kg)** | Canine | 72 | 30.6 (11.8, 44)^a^ | 1.02 | (0.99, 1.05) | 0.308 |
|  | Feline | 8 | 6.0 (4.6; 6.9)^a^ | 1.18 | (0.71, 1.96) | 0.639 |
| **Body composition** | Ideal | 53 | 10 (18.9%) |  |  |  |
|  | Overweight | 19 | 7 (36.8%) | 1.95 | (0.87, 4.40) | 0.174 |
| **Body condition score** |  | 72 | 5.7 (1.6) | 1.27 | (0.96, 1.69) | 0.160 |
| **Laterality** | Bilateral | 2 | 0 (0%) |  |  |  |
|  | Left | 34 | 8 (23.5%) |  |  |  |
|  | Right | 44 | 9 (20.5%) | 0.87^b^ | (0.38, 2.02) | 0.773 |
| **Forelimb or hindlimb** | Both | 4 | 2 (50%) |  |  |  |
|  | Forelimb | 26 | 3 (11.5%) |  |  |  |
|  | Hindlimb & pelvis | 1 | 0 (0%) |  |  |  |
|  | Hindlimb | 48 | 12 (25%) |  |  |  |
|  | Pelvis | 1 | 0 (0%) | 2.08 | (0.64, 6.72) | 0.257 |
| **Comminution** | No | 19 | 4 (21.1%) |  |  |  |
|  | Yes | 61 | 13 (21.3%) | 1.01 | (0.37, 2.74) | 0.983 |
| **Fracture site** | Calcaneus | 3 | 0 (0%) |  |  |  |
|  | Femur | 13 | 1 (7.7%) |  |  |  |
|  | Humerus | 8 | 1 (12.5%) |  |  |  |
|  | Metacarpal | 3 | 0 (0%) |  |  |  |
|  | Metatarsal | 2 | 0 (0%) |  |  |  |
|  | Multiple | 4 | 2 (50%) |  |  |  |
|  | Radius & ulna | 20 | 4 (20%) |  |  |  |
|  | Tibia & fibula | 27 | 9 (33.3%) |  |  |  |
| **Limb site** | Middle | 47 | 13 (27.7%) |  |  |  |
|  | Multiple | 4 | 2 (50%) | 1.81 | (0.61, 5.34) | 0.436 |
|  | Distal | 8 | 0 (0%) | 0.00 | (0.00, 0.00) | 0.993 |
|  | Proximal | 21 | 2 (9.5%) | 0.34 | (0.09, 1.39) | 0.160 |
| **Source of trauma (combined)** | Penetrating | 11 | 2 (18.2%) |  |  |  |
|  | Road traffic accident | 42 | 9 (21.4%) | 1.18 | (0.30, 4.69) | 0.833 |
|  | Trauma | 15 | 1 (6.7%) | 0.37 | (0.04, 3.55) | 0.413 |
|  | Unknown | 12 | 5 (29.4%) | 2.29 | (0.55, 9.49) | 0.322 |
| **History of infection** | No | 73 | 15 (20.5%) |  |  |  |
|  | Yes | 7 | 2 (28.6%) | 1.39 | (0.40, 4.88) | 0.661 |
| **Comorbidity** | No | 43 | 6 (14%) |  |  |  |
|  | Yes | 37 | 11 (29.7%) | 2.13 | (0.87, 5.20) | 0.092 |
| **Time to surgery (hours)** |  |  | 48 (18; 120)^a^ | 0.99 | (0.99, 1.00) | 0.348 |
| **Duration of anaesthesia (minutes)** |  |  | 265 (180; 555)^a^ | 1.00 | (1.00, 1.00) | 0.810 |
| **Duration of surgery (minutes)** |  |  | 149.4 (59.2)^a^ | 1.00 | (0.99, 1.00) | 0.902 |
| **Number of anesthetic events** | 1 | 68 | 15 (22.1%) |  |  |  |
|  | 2 | 10 | 1 (10.0%) |  |  |  |
|  | 3 | 2 | 1 (50%) | 1.01 | (0.35, 2.92) | 0.989 |
| **Total anaesthetic time (minutes)** |  |  | 265 (180, 850) | 1.00 | (1.00, 1.00) | 0.891 |
| **Surgical approach** | MIO | 15 | 4 (26.7%) |  |  |  |
|  | ORIF | 65 | 13 (20%) | 0.75 | (0.28, 1.98) | 0.615 |
| **Time from injury to antibiotic administration (minutes)** |  |  | 843.5 (1508.2) | 1.00 | (1.00, 1.00) | 0.814 |
| **Type of class of perioperative antibiotic** | Beta-lactam | 43 | 7 (16.3%) |  |  |  |
|  | 1st generation cephalosporin | 37 | 10 (27.0%) | 1.66 | (0.70, 3.92) | 0.304 |
| **Duration of post-operative antibiotics (weeks)** |  |  | 2.14 (0.71; 17.0)^a^ | 1.13 | (1.08; 1.19) | 0.046 |
| **Type of class of post-operative antibiotics** | Beta-lactam | 45 | 8 (17.8%) |  |  |  |
|  | 1st generation cephalosporin or tetracycline | 32 | 8 (25.0%) | 1.41 | (0.59, 3.35) | 0.495 |
| **Use of skin staples** | No | 34 | 6 (17.6%) |  |  |  |
|  | Yes | 46 | 11 (23.9%) | 1.36 | (0.56, 3.30) | 0.549 |
| **Wound closure** | Primary | 68 | 14 (20.6%) | 0.82 | (0.28, 2.44) | 0.73 |
|  | Other | 12 | 3 (25.0%) |  |  |  |
| **Duration of follow-up (weeks)** |  |  | 115.5 (164.7) | 1.00 | (1.00, 1.00) | 0.343 |
| **Post-operative infection** | No | 66 | 11 (16.7%) |  |  |  |
|  | Yes | 13 | 6 (46.2%) | 2.77 | (1.25, 6.15) | 0.045 |
|  | NA | 1 | 0 (0%) |  |  |  |
| ^a^ Median (range)  ^b^ Compares left and right (excluding bilateral) | | | | | | |

RR: Risk ratio, CI: Confidence interval, MIO: Minimally invasive osteosynthesis, ORIF: Open reduction and internal fixation
